# The Fear of Overfilling (FOF): A Clinically Significant Response to Digital Aesthetics and the Dermatologist's Imperative to Preserve Facial Identity

**DOI:** 10.1111/jocd.70652

**Published:** 2026-01-12

**Authors:** Rafael Rodrigo Crisanto de Oliveira

**Affiliations:** ^1^ Universidade Federal da Paraíba, Centro de Ciências da Saúde João Pessoa State of Paraíba Brazil

Hyaluronic acid (HA) fillers remain the cornerstone of contemporary aesthetic dermatology, offering reversibility, predictable outcomes, and the capacity to rejuvenate while maintaining core facial identity when anatomically guided [1, 2]. Alongside the evolution in technique and safety, a parallel sociocultural transformation driven by digital aesthetics has reshaped public perception of fillers. Within this landscape, two interconnected phenomena have emerged as central to modern clinical practice: Overfilled Face Syndrome (OFS) and its psychological counterpart, the Fear of Overfilling (FOF). This commentary proposes FOF as a conceptual framework and heuristic tool rather than a formally validated diagnostic entity, serving to understand a clinically significant response to the hypervisibility of distorted aesthetic outcomes in digital environments. Recognizing FOF as a structured psychocultural phenomenon is essential for improving communication, enhancing adherence, and guiding modern treatment planning.

FOF is conceptualized as a psychologically and culturally mediated fear of identity disruption, not merely of poor aesthetic results. It is crucial to distinguish this construct from Body Dysmorphic Disorder (BDD), which is characterized by a pathological internal distortion of self‐perception and an obsessive pursuit of correction for non‐existent or slight physical defects [3]. While BDD involves a persistent preoccupation with perceived flaws, FOF represents a rational, anticipatory concern regarding iatrogenic identity loss—the fear of “losing one's self” through clinical intervention. Furthermore, FOF is distinct from general procedural anxiety; while the latter is a physiological or psychological response to the medical act itself, FOF focuses specifically on the long‐term aesthetic and psychosocial consequences of over‐augmentation and the disruption of facial coherence. By articulating FOF as a distinct conceptual clinical construct, dermatologists are positioned as guardians of identity coherence amid an environment where unnatural aesthetic outcomes are simultaneously normalized and feared.

Modern patients frequently express concerns about looking “overdone” or “artificial,” worries heightened by the saturation of exaggerated outcomes on social media platforms [4, 5]. Digital filters, portrait‐mode elongation, and reshaping algorithms have collectively shifted internal norms of facial proportions. Although these tools promote idealized images, they paradoxically reinforce fear toward aesthetic procedures.

In this context, FOF emerges primarily as a fear of identity loss, representing a fundamental shift in patient psychology: Aesthetic decisions are increasingly based not only on traditional risk–benefit considerations but on the perceived threat to personal recognizability. This shift is further intensified by the paradox of enhancement: Progressive modifications—either through repeated filler sessions or repeated exposure to digitally exaggerated faces—gradually recalibrate the internal reference point for naturalness, a phenomenon often described as “aesthetic blindness”, where the perception of distortion is lost to the patient but heightened in the observer [4]. Hyperfilled faces become normalized within online ecosystems, while the visibility of distorted results simultaneously increases public anxiety [5]. This duality explains why some patients pursue aggressive contouring trends while others avoid any intervention, even when clinically appropriate.

The clinical relevance of FOF becomes evident when observing its effects on treatment‐seeking behavior and adherence. Many ideal candidates now defer minimally invasive procedures due to fear of an unnatural appearance, even preferring visible signs of aging over perceived aesthetic risk [1, 2, 4]. This avoidance reflects a broader cultural ambivalence: While demand for natural outcomes is at an all‐time high, the public's perception of what “natural” looks like has been distorted by digital aesthetics [6].

FOF, therefore, operates as a psychological barrier that must be addressed through structured communication, expectation management, and transparent demonstration of technique. At the same time, digital reputation has introduced new complexities into aesthetic decision‐making. Social media platforms disproportionately reward visually striking or exaggerated images, which often misrepresent routine clinical outcomes and create distorted perceptions of risk [4, 6]. As a result, patients may overestimate the prevalence of OFS, reinforcing FOF and challenging the therapeutic alliance. A dermatologist's digital presence must therefore prioritize restraint, identity preservation, and clinical transparency to counterbalance misinformation and algorithmic bias [7].

The influence of algorithm‐driven beauty standards on body image has been substantiated by literature linking repeated exposure to edited images with increased body dissatisfaction [6]. These findings support FOF as a rational response to environmental cues rather than an unfounded anxiety. Observational data from Google Trends reflect this rising global interest, with increased search volume for terms such as “dissolve filler” and “filler migration” [8]. While these metrics are proxies for public interest rather than clinical prevalence, they suggest a growing public consciousness regarding filler over‐accumulation.

Clinically, the demand for HA dissolution has grown, aligned with literature highlighting migration patterns, chronic accumulation, and long‐term structural changes [2, 5]. These dynamics reveal that FOF is anchored in observable clinical complications, such as OFS, which results from disproportionate volume distribution that disrupts facial harmony, effaces natural contours, and alters expressive dynamics [1]. Importantly, overfilling is not caused solely by excessive product. Contributing mechanisms include injection at incorrect depths, disregard for aesthetic subunits, reinjection before adequate tissue recovery, and filler migration over time [5, 9]. Chronic HA accumulation may impair lymphatic drainage and induce persistent edema, reinforcing that FOF is grounded in physiologic realities [2, 5].

The psychosocial consequences of OFS are amplified in the digital era, where viral images serve as emotional triggers. Some patients adopt hyper‐defensive aesthetic attitudes, requesting only the most conservative interventions, while others develop treatment‐induced aesthetic anxiety, where fear of overcorrection outweighs potential rejuvenation benefits [4, 5]. In this context, communication becomes a therapeutic intervention. High‐quality photography, dynamic facial analysis, and transparent discussion of reversibility (including hyaluronidase use) all serve to reduce FOF and rebuild trust [5, 9].

To address OFS and FOF simultaneously, an integrated, identity‐centered clinical framework is required, based on four essential pillars:

1. Advanced Anatomical Mastery With Dynamic Evaluation: Static assessment alone is insufficient. Evaluating patients during smiling, speaking, and brow animation helps identify subtle risks of expressive distortion and prevents structural incongruence, particularly relevant in patients expressing high levels of FOF [5].
2. Age‐Related Structural Prioritization: Emphasizing structural support over volume reduces the risk of superficial augmentation that disrupts natural contouring. Considering bone resorption patterns, fat compartment evolution, and ligamentous attenuation enables restoration without excess [2].
3. Bioregenerative and Non‐Volumizing Modalities: Biostimulators, polynucleotides (PDRN), and laser‐based therapies improve skin rheology and structural integrity without unintended volumetric expansion, making them ideal for patients exhibiting strong FOF [10]. Combined modalities—such as PDRN, retinoic acid, and targeted laser therapy—have shown efficacy in improving texture and pigmentation without contributing to volume‐related complications [11].
4. Dermatologic Ultrasound as an Emerging Best Practice: High‐resolution ultrasound provides real‐time visualization of filler location, chronic deposits, and migration pathways [1, 9]. As a valuable diagnostic adjunct, it enhances procedural precision while offering the “visual proof” required to decouple subjective anxiety from anatomical reality. By quantifying tissue status and mapping existing deposits, ultrasound serves as a transparent planning tool that effectively mitigates the fear of iatrogenic identity loss.

In conclusion the recognition of FOF necessitates a strategic shift from volume‐centric discussions to an identity‐preservation framework. Clinically, this involves a “de‐escalation” consultation approach that prioritizes the screening of aesthetic anxiety and emphasizes structural restoration over superficial augmentation. Treatment planning for patients with high FOF should favor multi‐session, incremental adjustments and the integration of volume‐neutral modalities to achieve rejuvenation without perceived facial distortion. Outcome assessment must also evolve beyond static photography, incorporating dynamic facial analysis and dermatologic ultrasound to provide objective evidence of tissue integrity and anatomical stability. By addressing the psychological barrier of identity loss through anatomical transparency and restorative techniques, the dermatologist can mitigate defensive patient attitudes and restore public confidence in safe, natural, and individualized rejuvenation, reaffirming their role not merely as an injector but as a steward of facial identity in the digital age.

## Ethics Statement

The author has nothing to report.

## Conflicts of Interest

The author declares no conflicts of interest.

## Data Availability

The data that support the findings of this study are available on request from the corresponding author. The data are not publicly available due to privacy or ethical restrictions.
